# Network pharmacology, molecular docking and *In vivo* validation reveal the neuroprotective effects of TT-TeMac™ against cholinergic dysfunction and hippocampal lesions

**DOI:** 10.1016/j.ibneur.2026.08.007

**Published:** 2026-08-10

**Authors:** Bruno Dupon Akamba Ambamba, Messanga me Ngo'o Jonathan, Akono Fama Yves Marc, Nyabissick Mondjiep Sandrine, Njayou Mbouangouore Ingrid Reine, Ngarchindi Emmanuel, Nkodo Abega Laurent, Njanjo Ejanmoua Merveille La Blonde, Ebogo Enyegue Françoise Alexandra, Fils Armand Ella, Damaris Enyegue Mandob, Judith Laure Ngondi

**Affiliations:** a Department of Biochemistry, Faculty of Science, University of Yaounde 1, Yaounde P.O. Box 812, Cameroon; b Center of Nutrition and Functional Foods, Yaounde P.O. Box 8024, Cameroon; c Department of Biological Sciences, Higher Teacher’s Training College, University of Yaounde 1, Yaounde P.O. Box 47, Cameroon; d Centre for Research on Medicinal Plants and Traditional Medicine, Institute of Medical Research and Medicinal Plants Studies, Yaounde P.O. Box 13033, Cameroon

**Keywords:** Network pharmacology, Molecular Docking, TT-TeMac™, Cholinergic dysfunction and memory loss

## Abstract

AD is a neurodegenerative disorder marked by progressive cognitive decline, particularly memory impairment, largely driven by cholinergic dysfunction. This study aimed to investigate the pharmacological basis and modes of action of the identified TT-TeMac™ compounds againsts cholinergic dysfunction associated with memory loss. TT-TeMac™ compounds targets which were identified by LC-MS were extracted from SwissTarget Prediction and PharmMapper, while cholinergic dysfunction-related targets were obtained from DisGeNET and GeneCards. Fifteen common targets were identified, with nine key targets highlighted by STRING, Cytoscape, and Venny analyses and confirmed by molecular docking using MOE. The *in vivo* validation used a rat model with scopolamine-induced cholinergic dysfunction (1 mg/kg bw/day, ip) for 7 days. Subsequently, behavioral (NOR and MWM), biochemical (AchE and BuhE) and histological (H&E and CV) analyses were performed. Seven compounds were identified in TT-TeMac™ (terminolic acid, sericic acid, arjunolic acid, gallic acid, ellagic acid, 3-O-methyl ellagic acid and 3,3′-di-O-methyl ellagic acid). Network pharmacological analysis showed that TT-TeMac™ acted on 15 common targets of which ACHE, IL6, TNF, SNCA, AKT1, SERPINE1, STAT3, ACE, and ALB were the pivotal genes. Also, docking studies confirmed the involvement of the target within the network with meaningful binding energies. Furthermore, TT-TeMac™ prevented cholinergic dysfunction associated with memory loss in rats by significantly reducing cholinesterase activity and protecting against morphological alterations and neuronal loss in the hippocampus. Our study shows that the ingredient TT-TeMac™ has a multi-targeted mode of action on protein targets involved in cholinergic dysfunction and counteracts this dysfunction in scopolamine-treated rats.

## Highlights

• Seven TT-TeMac™ compounds: terminolic, sericic, arjunolic, gallic, ellagic, 3-O-methyl and 3,3′-di-O-methyl ellagic acids.
• 09 key genes among 15 targets were linked to cholinergic dysfunction: ACHE, IL6, TNF, SNCA, AKT1, SERPINE1, STAT3, ACE, ALB.
• TT-TeMac™ preserves learning, memory and protects the hippocampus (CA1–CA3, DG) of the brains of scopolamine-treated rats.
• TT-TeMac™ modulates cholinesterase (AchE and BuchE) metabolism in scopolamine-treated rats.
• TT-TeMac™ may target multiple proteins involved in cognitive decline, offering an advantage over single-target treatments.

## Graphical Abstract

**Figure:**
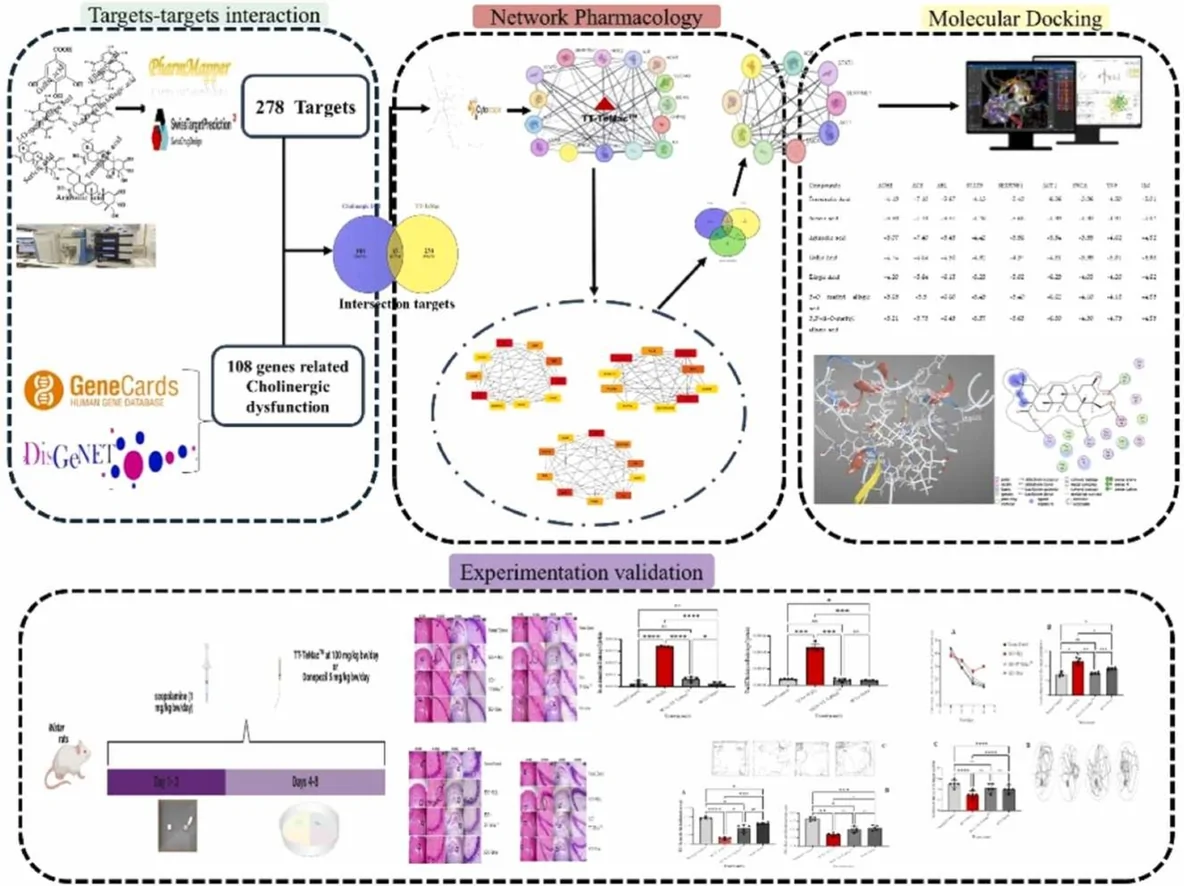

## 1 Introduction

The increase in life expectancy in recent decades has led to a rise of age-related diseases (Guo et al., 2022), including dementias. AD is the most common form and its prevalence is around 40% in people over 85 years of age (Alzheimer’s disease facts and figures, 2023). In the early stage of disease development, patients experience a decline in cognitive function and difficulty remembering recent events (short-term memory loss) (Reiss et al., 2022). During the progression of the disease, patients encounter difficulties in speech and cognitive thinking, accompanied by long-term memory loss. There is a strong relationship between cognitive decline and the depletion of the neurotransmitter acetylcholine in the brain of AD patients (Zuin et al., 2022).

Reason why cholinergic hypothesis has been central to the development of anti-Alzheimer's drugs (Zuin et al., 2022). The advances in the pathophysiological understanding of AD have elucidated the links between cholinergic hypothesis and other pathological mechanisms, including the formation and accumulation of Aβ, hyperphosphorylation of tau protein and neuroinflammation (Monteiro et al., 2023, Hampel et al., 2021). Current treatments for Alzheimer's disease primarily target its symptoms rather than the disease itself. Cognitive function can be temporarily improved by medications such as memantine, galantamine, rivastigmine, and donepezil. Immunotherapies like Aducanumab, Lecanemab and Donanemab have recently demonstrated the ability to slow the progression of the disease somewhat by reducing amyloid plaques, but their use remains limited due to adverse effects and access issues. Current research focuses on identifying anti-Alzheimer's agents (disease-modifying therapies) capable of preventing mild cognitive decline and slowing its progression. This is particularly crucial given the promising initial results of research focused on early diagnosis of the disease (Hajjo et al., 2022).

Polypharmacology is an approach more suited to the pathological complexity of AD, hoping to overcome the limitations of current treatments. This is why up to 03 agents described as multi-targeted have entered clinical trials in January 2025 in the AD drug development pipeline (Cummings et al., 2025). Recent advances in life sciences have highlighted the growing importance of network pharmacology in modern research. Furthermore, it integrates the principles of systems biology to explore biological networks and identify key signalling nodes that may serve as potential therapeutic targets with diverse modes of action. Coupled with molecular docking approaches, these models can predict the mode of action of candidates on specific targets (Jabalia et al., 2021). Experimental validation is an essential step in the translation of hypotheses using a bioinformatics approach, particularly in the context of complex diseases requiring a multitarget and multifactorial approach such as AD. Since the discovery of AD, several animal models have emerged, but the pharmacological model using scopolamine makes it possible to better mimic the cholinergic dysfunction associated with AD (Tang, 2019).

Bioactive compounds from plants are increasingly sought after for their anti-Alzheimer properties (Chen et al., 2021). Among these, terpenoids and tannins are being increasingly exploited in the search for anti-Alzheimer's ingredients due to their synergistic and complementary effects on cholinergic dysfunction (Yoo and Park, 2012; Hussain et al., 2019). In this context, several natural products such as green tea extract and rosemary extract, which are rich in terpenoids and tannins, have demonstrated neuroprotective effects in the preclinical phase (Malar et al., 2020; Oresanya and Orhan, 2024).

Recently, we showed that *Terminalia macroptera* tannin-rich extracts (TeMac™) and glycosyl terpenoid-rich fractions (GT-TeMac™), each prevent hippocampal alterations and cholinergic dysfunction in scopolamine-treated rats (Ambamba et al., 2025a, Ambamba et al., 2025b). Adey et al. (2021) showed that the ethyl acetate fraction of *Terminalia macroptera* combines tannins and terpenoids. In addition, a study by Ior et al. (2021) showed that this fraction administered at a dose of 100 mg/kg body weight, attenuated psychotic symptoms in mice. The objective of this work is to study the effect of the combined tannin-terpenoid fraction (TT-TeMac™) on the prevention of scopolamine-induced cholinergic dysfunctions and neuronal alterations. This by integrating phytochemical approaches to identify bioactive compounds, network pharmacology to predict potential targets involved in cholinergic dysfunction and identified compounds, molecular docking to assess compound-target interactions, and an *in vivo* model for experimental validation.

## 2 Materials and Methods

### 2.1 Drugs, chemicals and kits

***Drugs:*** Scopolamine was purchased from Cooper (Copper, France) and donepezil from Biwole Abondo Pharmacy (Yaounde, Cameroon).

***Chemicals:*** All the chemicals used were of analytical grade and purchased from Sigma Co., Louis, MO, USA.

### 2.2 Plant material, harvesting and processing

*Terminalia macroptera* (*T. macroptera*) barks were collected in Baligui in January 2021 (Centre, Cameroon) and identified at the National Herbarium of Cameroon, Yaoundé (in comparison with specimen N° 43688/HNC). The barks were washed, dried, and ground to obtain a powder, which was stored in opaque vials. This powder was used to prepare the TT-TeMac™.

### 2.3 Preparation of the TT-TeMac™

It was carried out by optimizing the protocol of Adey et al. (2021). 1000 g of bark powder was extracted with 3.5 L of methanol. The extract was concentrated using a rotary evaporator, yielding a blue-black solid mass of the methanol bark extract. A mass of 184.2 g of extract with a yield of 18.42% was obtained. The *T. macroptera* bark extract (100 g) was reconstituted in 300 mL of distilled water and subjected to liquid-liquid partition. The reconstituted extract was placed in a separating funnel, and 300 mL of hexane was added sequentially as a 1:1 (v/v) solution and stirred. The sample was allowed to stand for 60 min in the separating funnel until a thin separation line appeared, clearly indicating the supernatant above the sediment before desorption. This process was repeated until exhaustion, and the hexane fraction was collected and concentrated using a rotary evaporator. The same procedure was repeated sequentially with ethyl acetate and n-butanol to obtain the ethyl acetate fraction, n-butanol fraction and residual water fraction, respectively. The concentrated ethyl acetate fraction constituted the TT-TeMac™ and was used for further work.

### 2.4 Determination of total terpenoid content

Terpenoids were measured using the method of Ghorai et al. (2012). Two hundred microliters (200 µL) of TT-TeMac™ (4 mg/mL) were added to 1.5 mL of chloroform. The mixture was homogenized using the vortex. Then 100 µL of sulfuric acid 6 N was added to each tube (white, trial and standards). The mixture was cooled using an ice pack for 10 min. Absorbance was measured at 538 nm after 2 h of incubation at room temperature in the dark. Calibration was performed using oleanolic acid (0–1000 μg/mL).

### 2.5 Determination of total tannins content

The method of Medini et al. (2014) was used to estimate the tannin of TT-TeMac™. 1 mL of extract (4 mg/mL) was mixed with 5 mL of the working solution (50 g of vanillin plus 4 mL of 1 N HCl in 100 mL of distilled water) and the mixture was incubated at 30°C for 20 min. Absorbance was read at 500 nm against blank. Tannic acid (0–1000 μg/mL) was used as standard.

### 2.6 Identification of terpenoids and Tannins TT-TeMac™

Terpenoids and tannins were identified by liquid chromatography-mass spectrometry (LC-MS) as previously described by Teixeira et al. (2016). Raw LC-MS spectra data were converted with MSconvert, processed and analyzed with MZmine 2.53 software for Windows.

### 2.7 Computational Study

#### 2.7.1 Network pharmacology analysis

##### 2.7.1.1 Identification of potential targets of active compounds

Structural on active compounds of TT-TeMac™ were drawn from chemdraw Professional 15.0 (version 15.0, PerkinElmer Informatics, USA). Potential human target genes of TT-TeMac™ compounds were identified using the SwissTarget Prediction (http://swisstargetprediction.ch/) and PharmMapper (http://www.lilab-ecust.cn/pharmmapper/) databases. Duplicate genes were removed using Microsoft Excel to obtain the final list of target genes.

##### 2.7.1.2 Identification of potential targets of cholinergic dysfunction

Potential human target genes associated with cholinergic dysfunction were harvested from online databases, including GeneCards (http://www.genecards.org/), DisGeNET (https://www.disgenet.org/).

The keyword “Cholinergic Dysfunction” was used to perform the search, and duplicate genes were removed to obtain a comprehensive list of genes associated with human cholinergic dysfunction.

##### 2.7.1.3 Construction and analysis of the protein-protein interaction (PPI) network

Common target genes were obtained using Venny 2.1 (https://bioinfogp.cnb.csic.es/tools/venny/). Common target genes were submitted to the STRING database to construct a PPI network. To ensure data reliability, a confidence score ≥ 0.4 was set for target acquisition with “Homo sapiens”. PPI results were imported into Cytoscape software for network generation and analysis. Significant genes were identified using the CytoHubba plugin, based on three parameters: maximum clique centrality (MCC), maximum neighbourhood component (MNC), and degree. The ten most significant genes were identified based on their scores. The data was imported into a Venn and an intersection was constructed to identify likely hub genes in the network.

#### 2.7.2 Molecular Docking

MEO (version 2014.0901, Chemical Computing Group Inc., Montreal, QC, Canada) software was used to analyse the interactions between TT-TeMac™ compounds and the identified hub targets. The three-dimensional structures of the target proteins; were obtained from the RCSB Protein Data Bank in PDB format based on human origin and their high resolutions.

The docking study was completed carried out as such; (1) water molecules were removed from the protein structure; (2) hydrogen atoms with standard geometry were added to the structure, broken bonds were reconnected, the potential was fixed; (3) large sites in the enzyme structure were identified using the MEO Alpha Site Finder, and dummy atoms were generated from the resulting alpha spheres; (4) the interaction of the ligand with the active sites of amino acids was analysed. The ligand binding affinity was evaluated using the scoring function; dock function (S, Kcal/mol) created by the MOE 2014 software (Al-Karmalawy et al., 2021). Active ligands with the highest docking score have the most negative values.

### 2.8 Experimental animals

To validate the *in silico* results, an experimental study was carried out on male *Wistar* rats treated with scopolamine

#### 2.8.1 Ethical approval

Animals were treated following the guidelines of the ethical committee of the University of Yaoundé 1 (BTC-JIRB2024–101) and the Guide of the Care and Use of Laboratory Animals (8th edition). The number of animals has been reduced to the bare minimum and the protocols have been optimized to limit animal suffering.

#### 2.8.2 Experimental design

Twenty (20) male Wistar rats, weighing between 230 and 240 g, were obtained from the animal house of the Laboratory of Nutrition and Nutritional Biochemistry, Department of Biochemistry, Faculty of Science, University of Yaoundé 1. The animals were housed under controlled conditions with a constant temperature of 25 ± 2 °C, relative humidity of 50 ± 5%, and a 12/12-hour light/dark cycle. They had free access to food and water.

Animals were divided into 4 experimental groups of 5 rats each and treated as follows:

− (1) Normal Control group received distilled water (5 mL/kg bw/day; po)
− (2) SCO + H_2_O group received scopolamine (1 mg/kg bw/day, ip) and distilled water (5 mg/kg bw/day; po)
− **(**3) SCO + TT-TeMac™ group received scopolamine (1 mg/kg bw/day; ip) and TT-TeMac™ (100 mg/kg BW/day; po)
− (4) SCO + Done group received scopolamine (1 mg/kg/bw/day; ip) and donepezil (5 mg/kg bw/day; po).

Scopolamine was administered daily by intraperitoneal injection (1 mg/kg b.w) for 7 days to induce cognitive decline. The TT-TeMac™ and Donepezil were administrated by oral intubation through an esophageal tube 60 min after scopolamine injection. TT-TeMac™, donepezil, and distilled water were administered at a dose of 5 mL/kg b.w. The novel object recognition test was assessed on days 1, 2 and 3; and Morris Water Maze test on days 4, 5, 6, 7 and 8.

#### 2.8.3 Animal Sacrifice

At the end of the study, after a 12-hour fast, the rats were deeply anesthetized with ether, blood was collected by cardiac puncture, until the animals were euthanized by exsanguination, and their brains were carefully removed after cervical incision. Their brains were weighed and part was used to prepare homogenates and the other part to perform histopathological examinations.

At the time of the study, the use of ether had been approved (BTC-JIRB2024–101) by the Institutional Ethics Committee of the Faculty of Sciences at the University of Yaoundé 1. This ethics committee follows the guidelines of the National Committee for the Protection and Use of Animals regarding animal euthanasia. However, the researchers acknowledge that the use of ether is no longer recommended and that safer and more humane methods will be used in future studies**.**

### 2.9 Behavioural analysis

#### 2.9.1 Novel Object Recognition (NOR)

The novel object recognition test is used to determine learning, memory, recognition. In this study, the NOR test was performed in an Open Field device following the protocol adapted by Djiogue et al. (2018). The test consisted of 3 phases: habituation or pre-training, training, and trial or testing. During habituation phase, took place on the first day, each animal was placed in the field to explore the empty device box for 5 min and thereafter returned in the initial cage. During the training phase, animals were placed in the field for 5 min to explore two identical objects. The test was performed on the second day, 3 h after the training phase, to assess short-term memory, and on the third day, 24 h after training, to assess long-term memory. Exploration was defined as when the rat moved its snout toward or touched an object. The time spent exploring familiar and novel objects was recorded using a video recording system consisting of a camera positioned above the workspace and connected to a computer. After each trial, the box and objects were cleaned with a 70% ethanol solution to prevent any disturbance. The videos were analysed with the Any-Maze 7.1 software. The recognition index (RI) at short, and long-term memory was obtained. RI= (time spent exploring the new object) / (time spent exploring the new object +time spent exploring the familiar object)

#### 2.9.2 Morris Water Maze (MWM)

The MWM test provides an accurate and reproducible measure used to evaluate spatial learning and memory; as well as hippocampal damage (Morris, 1984). In the present study, the MWM test was performed in a black circular tank (diameter 120 cm × height 50 cm) half filled with water and divided into four equal quadrants. A black drainage platform was placed in a fixed position (south quadrant of the apparatus), submerged 1.0 cm below the surface of water. The test included a 4-day acquisition phase and a retention phase on day 5. The acquisition phase consisted on three trials per day with 15-minute break between trials. At the end of each trial, the animals were properly cleaned and returned to their home cages. During the acquisition trial, animals were left to explore the pool searching for the hidden platform. Once the animal located the platform, it was left there for 10 s. However, if an animal failed to reach the platform within 60 s it was guided there and kept for 10 s. During the retention phase, the platform was removed, and each rat was placed into the pool at one of the fixed targets facing the target quadrant and had 60 s to swim. All the parameters were recorded using a video recording system with a camera placed above the pool and connected to a computer. The videos were analysed with the Any-maze 7.1 software. The latency time (s) to reach the exact position of the platform and the number of entries in the target quadrant of the platform were expressed.

### 2.10 Histopathological examination

One of the hemispheres of brain of each rat was fixed in 10% formaldehyde, embedded in kerosene, cut (5 µm sections in the coronal plane), and processed for hematoxylin-eosin and Cresyl violet staining using standard procedures (Garman, 2011). Brain tissue damage was assessed in the hippocampal formation using a microscope connected to a camera (Axioskop 40, Zeiss, Hallbermoos, Germany). In addition, neurons in Cornus Ammonis (CA) regions 1, 2, 3 (CA1, CA2, CA3), and Dentate gyrus (DG) were counted using Image J software (version 1.52a).

### 2.11 Preparation of brain homogenate

The brain tissues were homogenized in 10% (w/v) ice-cold phosphate buffer saline, and centrifuged at 1500*g* for 10 min at 4°C. The brain homogenate (the supernatant) was immediately collected and stored at - 80°C for the brain cholinergic transmission evaluation.

### 2.12 Acetylcholinesterase (AChE) and Butyrylcholinesterase (BuChE) activities assays

Brain AChE and BuChE enzyme activities were determined using Ellman’s method. Acetylthiocholine iodide/S-butyrylthiocholine chloride was used as a substrate for AChE/BuChE. Thiocholine, produced forms a yellow complex with 5,5-dithiobis (2-nitrobenzoic acid). The absorbance was measured at 412 nm. The results are expressed in µmol/min/mg of protein (Ellman et al., 1961).

### 2.13 Total proteins determination

Protein estimation was carried out by the method described by Lowry et al. (1951).

### 2.14 Statistical analysis

GraphPad Prism software version 10.2.2 was used for statistical analysis. Normality test was confirmed by Shapiro-Wilk test, one-way analysis of variance (ANOVA) with Tukey test was performed for comparison between groups. Results are expressed as mean value ± standard deviation (n = 5 for *in vivo* tests). P values < 0.05 were considered significant.

## 3 Results

### 3.1 Tannins and Terpenoids content of TT-TeMac™

The level of tannins and terpenoids in the extract were 168.33 ± 8.1 mgEqAT/g TT-TeMac™ and 345.66 ± 3.16 mgEqOA/g TT-TeMac™, respectively.

### 3.2 Characterization and identification of terpenoids and tannins of the TT-TeMac™ by LCMS

The results of the LC-MS analysis are presented in Fig. 2 (chromatogram) and Table 1 (mass spectra).

**Fig. 1:**
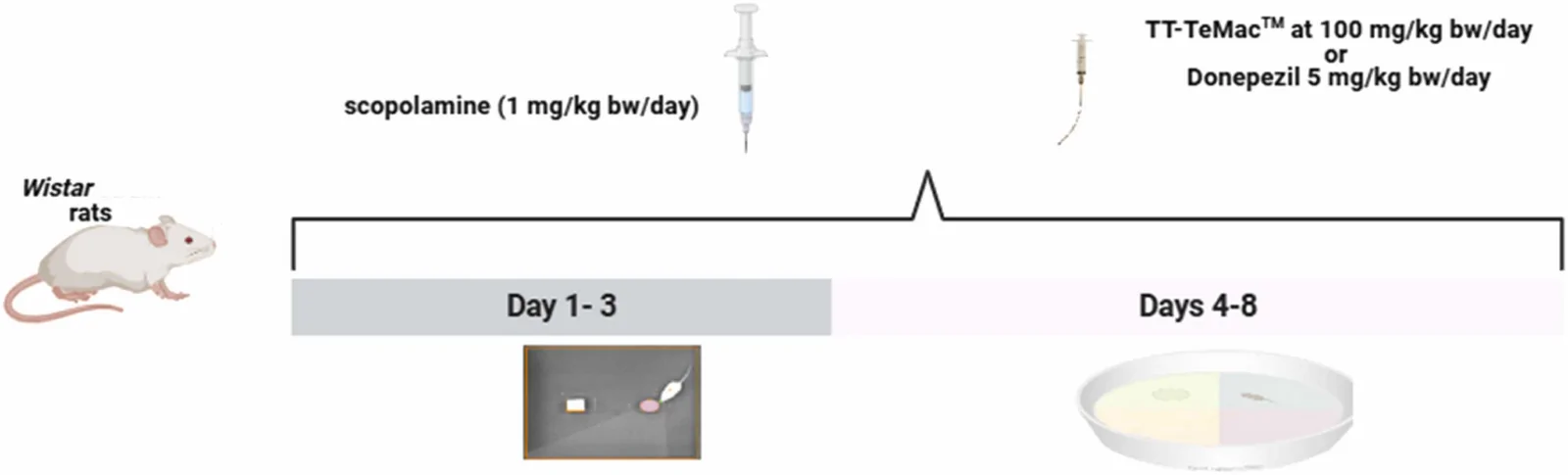
Experimentation design.

**Fig. 2:**
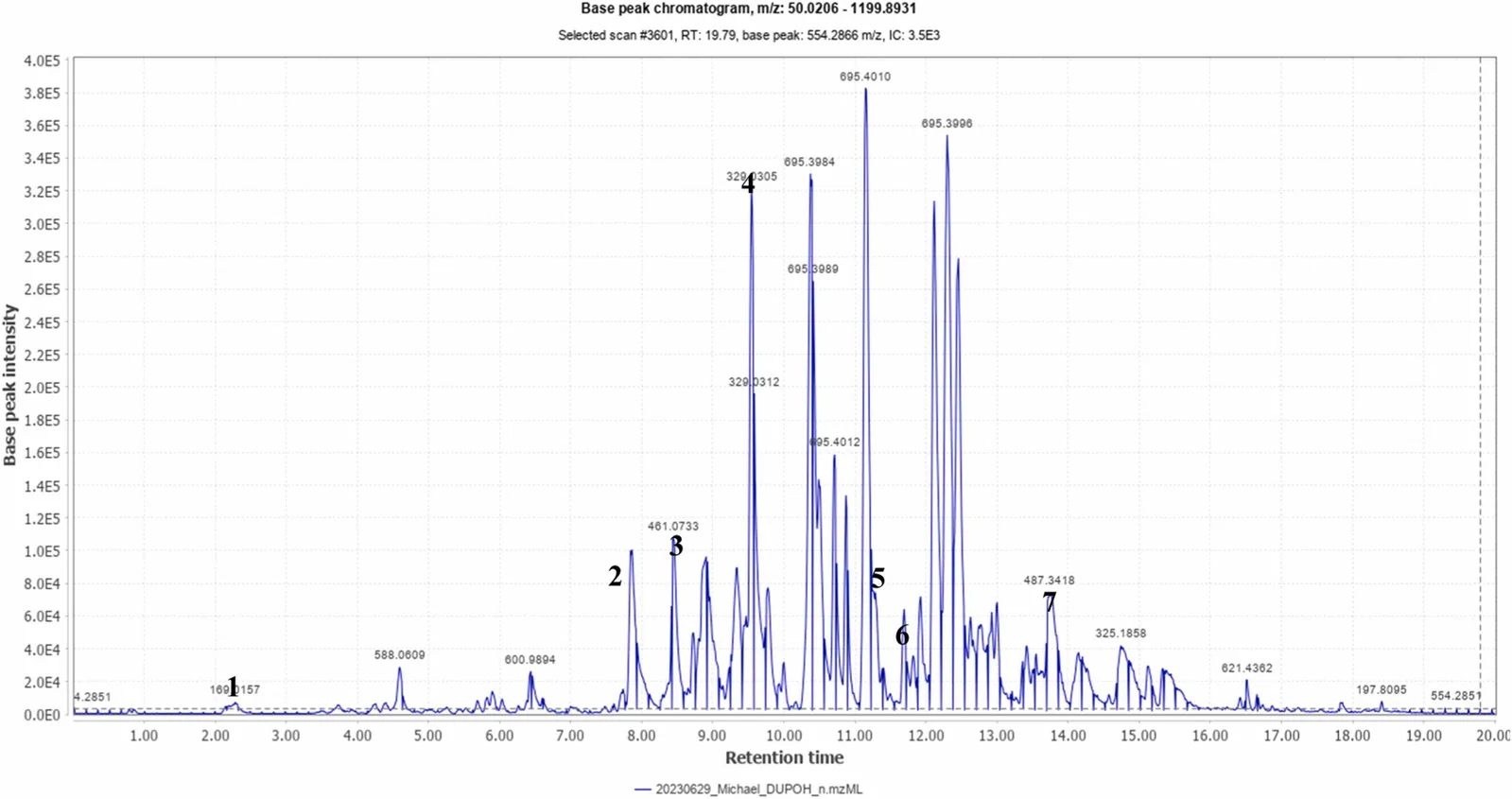
Chromatographic profile of TT-TeMac™.

**Table 1:** Compounds identified, molecular ions and their mass spectra.

#### 3.2.1 Chromatogram of TT-TeMac™

LC analysis of the combined fraction of terpenoids and tannin showed the presence of peaks with a retention time of 0–20 min (Fig. 2).

#### 3.2.2 Mass Spectra of compounds identified of TT-TeMac™

The MS allowed to identify seven compounds: Terminolic Acid, Sericic acid, arjunolic acid, Gallic acid, ellagic acid, 3-O methyl ellagic acid and 3,3’-di-O-methyl ellagic acid) (Table 1).

### 3.3 Target Identification

**Fig. 3:**
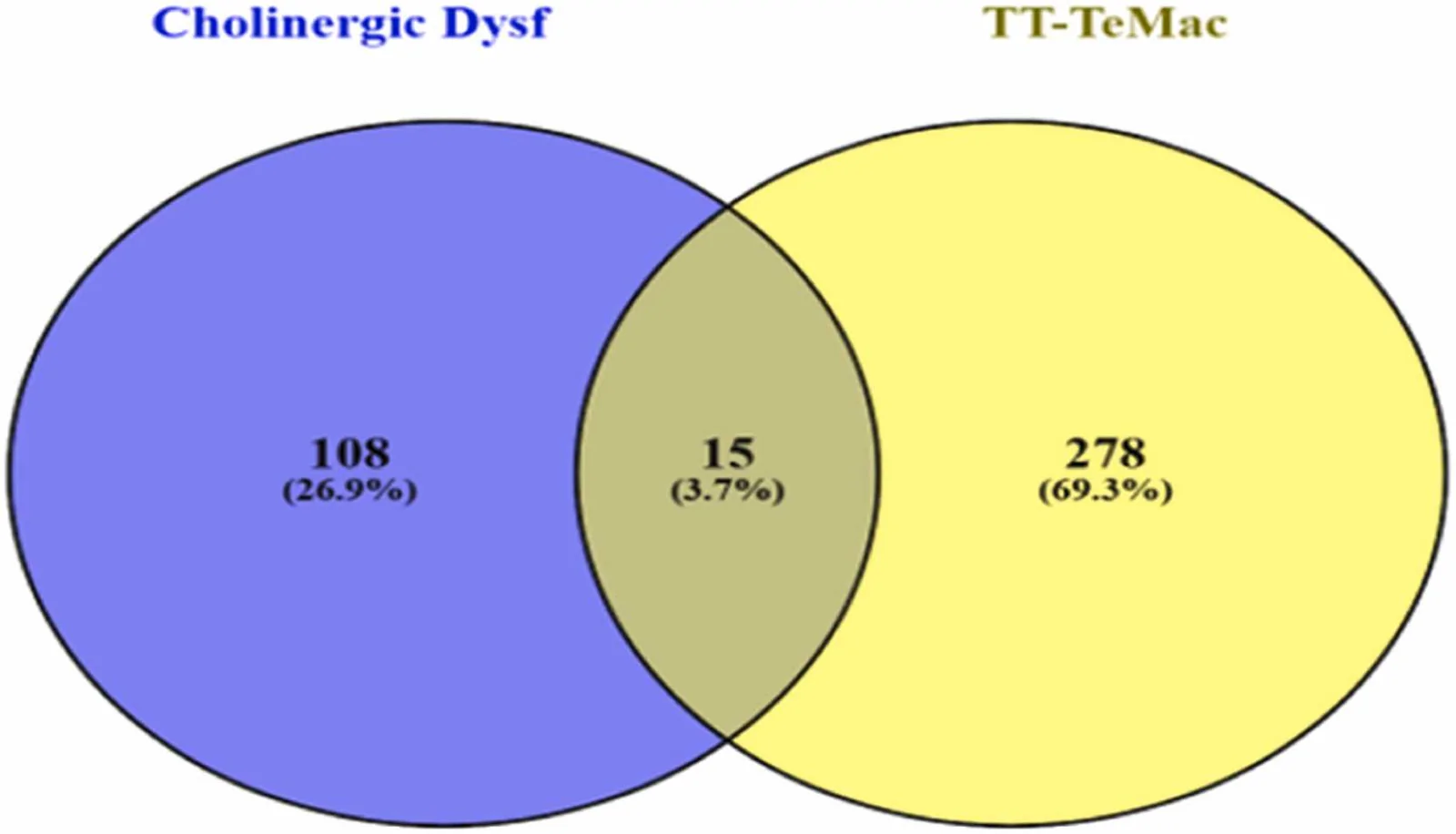
Venn diagram showing potential targets between TT-TeMac™ and cholinergic dysfunction targets.

A total of 293 TT-TeMac™ target genes and 123 genes associated with cholinergic dysfunction were identified, and a total of 15 genes were identified as intersection genes (Fig. 3).

**Fig. 4:**
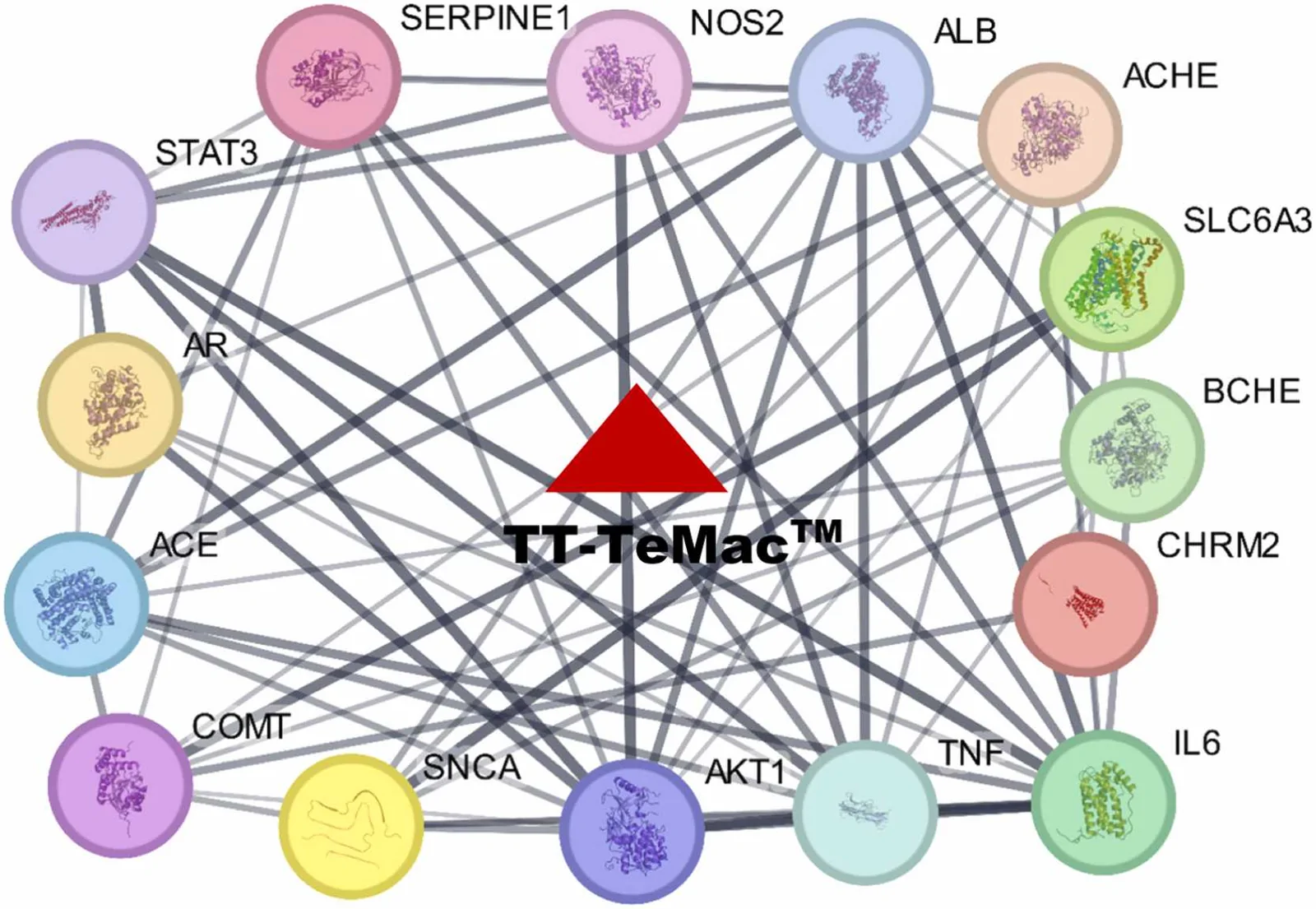
Compound-target network of TT-TeMac™ against cholinergic dysfunction generated by Cytoscape software. The nodes represent the interacting target genes of cholinergic dysfunction and the triangle represents the node of TT-TeMac™. SERPINE1: Serpin Family E Member 1; NOS2: Nitric-oxide synthase inducible; ALB: Albumin; ACHE: Acetylcholinesterase; SLC6A3: Dopamine transporter; BCHE: ButyrylCholinesterase; CHRM2: Muscarinic acetylcholine receptor M2; IL6: Interleukin−6; TNF: Tumor necrosis factor; AKT1: RAC-alpha serine/threonine-protein kinase; SNCA: Alpha-synuclein; COMT: Catechol O-methyltransferase; ACE: Angiotensin-converting enzyme; AR: Androgen receptor; STAT3: Signal transducer and activator of transcription 3.

**Fig. 5:**
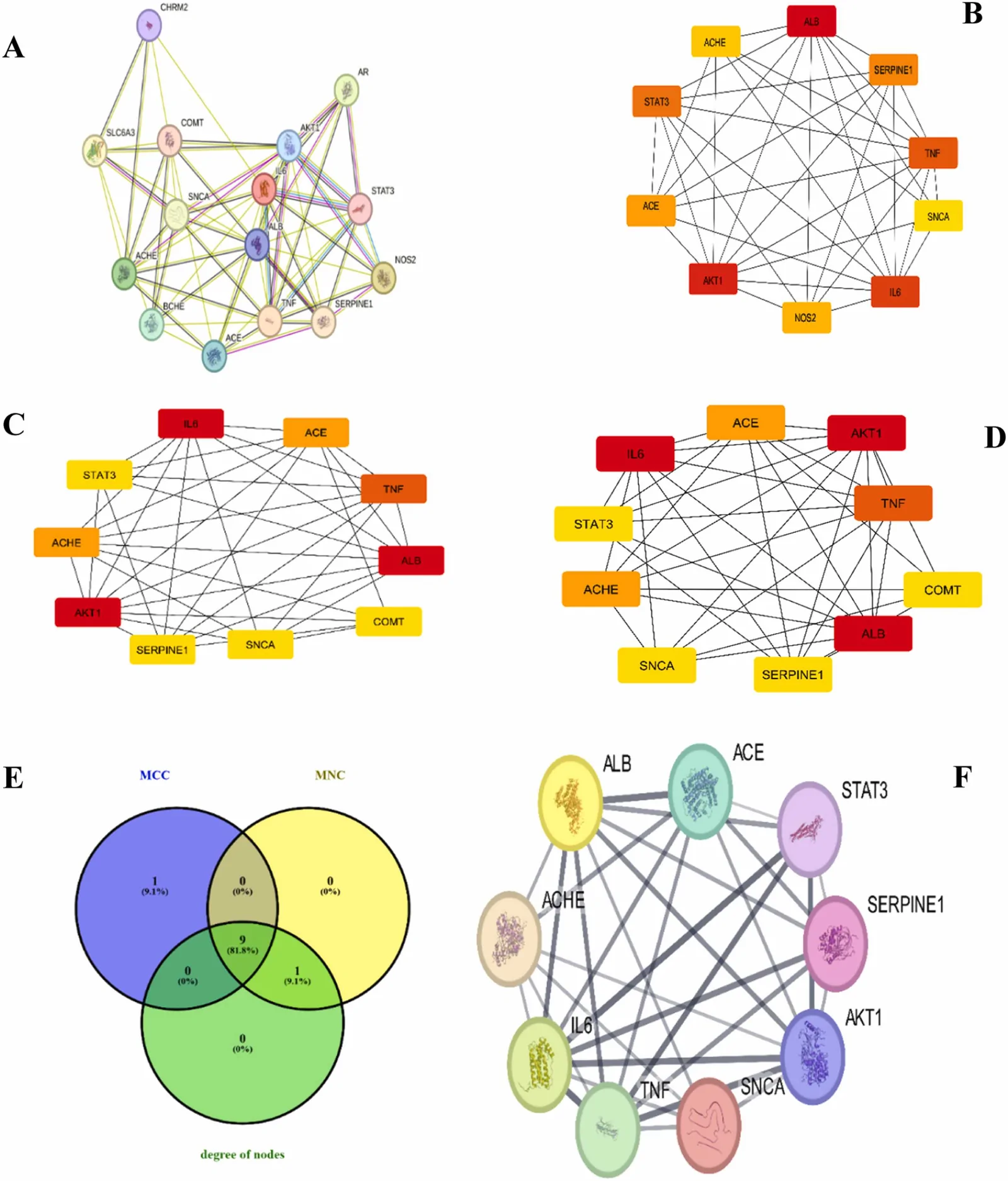
(A) Protein-protein interaction networks are constructed using the STRING database. The top ten hub gene networks of TT-TeMac™ against cholinergic dysfunction are obtained by integrating three algorithms: (B) Maximum Clique Centrality (MCC), (C) Maximum Neighborhood Component (MNC), (D) Node Degree. (E) Venn diagram illustrates nine hub genes. (F) Target network of nine hub genes generated by Cytoscape software.

### 3.4 TT-TeMac™-target network constructions

The compound target network consists of 15 compound target nodes, one active node of TT-TeMac™, and 126 edges. In the network, the circles represent the target proteins of cholinergic dysfunction, while the triangle denotes TT-TeMac™. This clearly demonstrates the multi-target nature of TT-TeMac™ against cholinergic dysfunction associated with memory loss. Therefore, we not only observed the relationship between TT-TeMac™ and cholinergic dysfunction, but also discovered the potential pharmacological effects of TT-TeMac™ from this network.

### 3.5 Top ten hub gene networks of TT-TeMac™ against cholinergic dysfunction

The figure shows the protein-protein interaction networks constructed using the STRING database. The top ten hub gene networks of TT-TeMac™ against cholinergic dysfunction are obtained by integrating three algorithms (maximum clique centrality (MCC), maximum neighbour component (MNC), node degree). The Venn diagram illustrates nine hub genes and the target network of nine hub genes generated by Cytoscape software. The nine hub genes include ACHE, IL6, TNF, SNCA, AKT1, SERPINE1, STAT3, ACE, and ALB.

### 3.6 Details of protein targets in the PDB database

Table 2 shows the details of protein targets identified in the Protein Data Bank (PDB), along with their respective PDB IDs, resolution, and R values obtained by X-ray diffraction except SNCA (Electron Microscopy). Target proteins include Acetylcholinesterase (ACHE), Angiotensin Converting Enzyme (ACE), tumor necrosis factor (TNF), albumin (ALB), Signal transducer and activator of transcription 3 (STAT3), Plasminogen activator inhibitor−1 (SERPINE1), protein kinase Akt (AKT1), alpha-synuclein (SNCA) and Interleukin−6 (IL6). Resolution values range from 1.80 to 2.98 Å.

**Table 2:** Details of the protein targets in the PDB database.

Targets name	PDB ID	Resolution (Å)	R-Value Free	R-Value Work
ACHE	4EY4	2.16	0.22	0.18
ACE	1O8A	2.00	0.22	0.18
ABL	1AO6	2.5	0.28	0.21
STAT3	6NJS	2.70	0.25	0.20
SERPINE1	1LJ5	1.80	0.24	0.19
AKT1	6S9W	2.30	0.26	0.21
SNCA	6SSX	2.98	/	/
TNF	2AZ5	2.10	0.27	0.22
IL6	1ALU	1.90	0.27	0.21

### 3.7 Molecular Docking results

Table 3 presents the molecular docking scores of the identified TT-TeMac™ compounds with the major targets involved in cholinergic dysfunction. The highest MOE scoring function was applied to the tested compounds to evaluate their binding affinities. The compounds showed good binding affinity values ranging from −3.39 kcal/mol (gallic acid) to −7.4 kcal/mol (Arjunolic acid). The considerable RMSD value of < 2 indicates the high stability of these compounds in the binding site (SM 2).

**Table 3:** Binding energy between the compounds of TT-TeMac™ and prioritized cholinergic dysfunction target proteins.

Compounds	ACHE	ACE	ABL	STAT3	SERPINE1	AKT1	SNCA	TNF	IL6
Terminolic Acid	−4.19	−7.10	−5.67	−4.13	−5.49	−6.36	−3.96	−4.50	−5.01
Sericic acid	−5.59	−7.19	−5.31	−4.26	−5.66	−4.99	−4.38	−4.91	−4.62
Arjunolic acid	−5.07	−7.40	−5.45	−4.42	−5.96	−5.34	−3.99	−4.62	−4.82
Gallic Acid	−4.74	−4.64	−4.56	−4.32	−4.37	−4.81	−3.39	−3.81	−3.98
Ellagic Acid	−4.20	−5.64	−6.15	−5.25	−5.02	−6.29	−4.05	−4.20	−4.62
3-O methyl ellagic acid	−3.65	−5.5	−6.60	−5.43	−5.40	−6.62	−4.10	−4.18	−4.83
3,3’-di-O-methyl ellagic acid	−5.21	−5.73	−6.43	−5.57	−5.63	−6.80	−4.30	−4.79	−4.89

Table 4 and SM1 show the profiles and different interactions of the compounds of interest with the targets. The compounds interacted with important residues of the target active pockets.

**Table 4:** Interaction profile of amino acids in the active pockets of target proteins with TT-TeMac™ compounds.

Compounds	AChE	ACE	ABL	STAT3	SERPINE1	AKT1	SNCA	TNF	IL6
Terminolic Acid	Trp86, Asp74, Tyr341, Tyr124	Arg124, Asn66, Ser335, Asn70	Lys395, Cys448	Cys259	Asp222	Gln59, Ala58	Gln79	Leu43	Asp160, Arg104
Sericic acid	Tyr72, Gly120, Glu202	His 410, Asn70	Ala201, Asn298, Lys614, Lys395	Glu238	Glu242, Lys241	Ser56	Gly36, Leu38	Trp28	Arg104
Arjunolic acid	Val73, Gly448, Glu202, His447	Asn70, Asp358	Lys195	Arg246, Val136	Arg157	Arg200	Tyr39	Arg32	Glu42, Lys46
Gallic Acid	Phe338, Tyr124	Asn66, Glu143	His242	Cys259	Val274	Thr211	Gln79	Arg32, Trp28	Arg104
Ellagic Acid	Tyr124, Glu202	Asn374, Asp377, Glu162	Ala291, His242	Cys259	Leu272	Lys268, Thr211, Trp80	Lys80	Gln25	Arg104, Thr43
3-O-methyl ellagic acid	Tyr337, Trp86	Tyr62, Ser355	Ala291, Trp214	Gln141, Asn257	Val274	Tyr211, Trp80	Leu38	Gln25	Arg104, Thr43
3,3’-di-O-methyl ellagic acid	Glu202, Trp86	Tyr62, Ser355	Ser287	Val137	Phe358, Val274	Gly294	Lys80	Asp45	Thr43

### 3.8 TT-TeMac™ prevents non-spatial recognition learning and memory in the NOR test

**Fig. 6:**
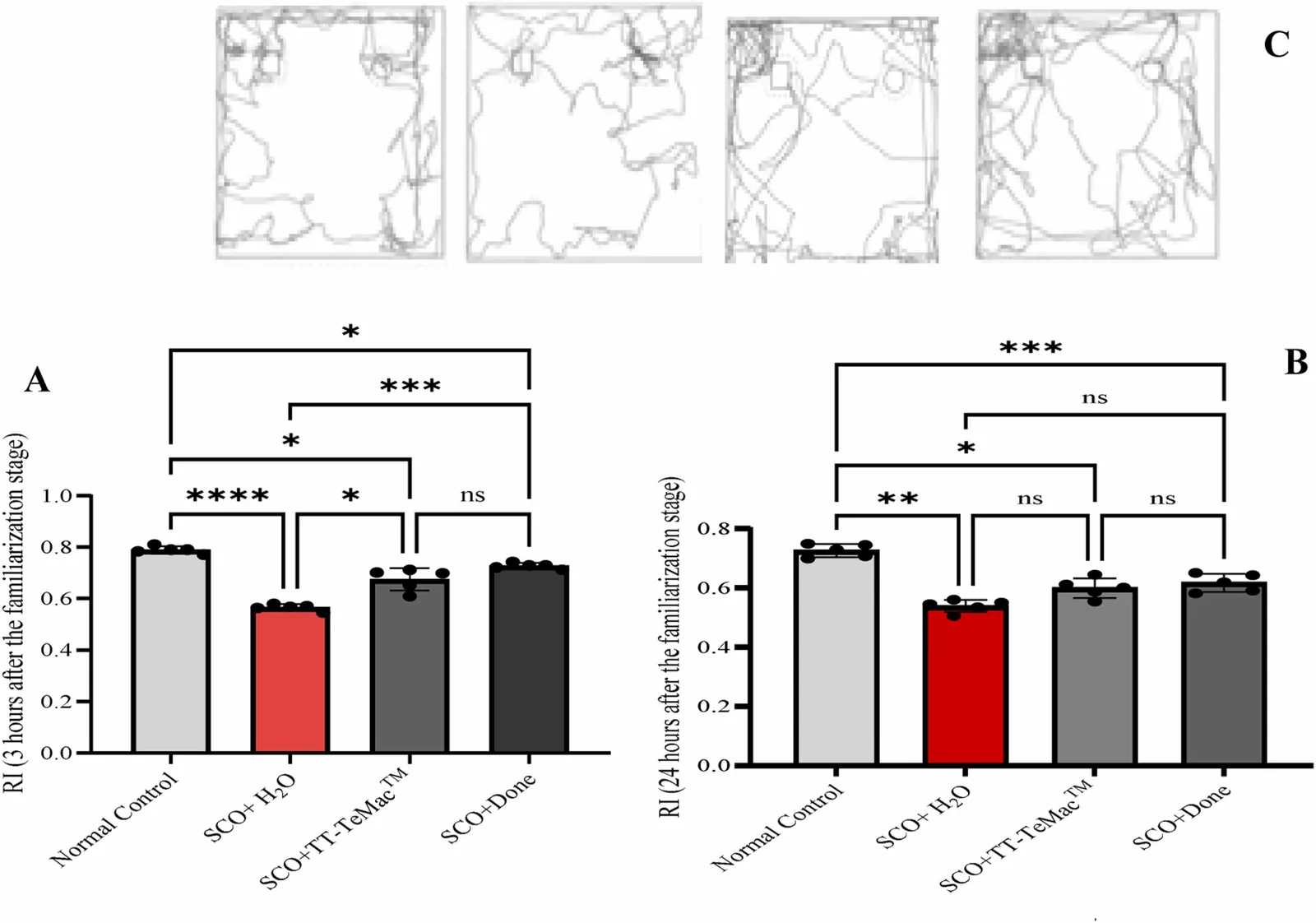
TT-TeMac™ prevents scopolamine-induced short- and long-term nonspatial learning and memory impairments in rats. (a) *Short Term Memory IR (3 h after familiarization). (B) long term Memory IR (24 h after familiarization). (C) Representative tracks of rats moving during the test. **Normal Control (n = 5)**: Rats treated with distilled water; **SCO + H***_***2***_***O (n = 5)**: Rats treated with 1 mg/kg body weight/day scopolamine + distilled water; **SCO + TT-TeMac***^***TM***^***(n = 5)**: Rats treated with 1 mg/kg body weight/day scopolamine + 100 mg/kg body weight/day TT-TeMac*^*TM*^*; **SCO + Done (n = 5)**: Rats treated with 1 mg/kg body weight/day scopolamine + 5 mg/kg body weight/day donepezil.* One-way ANOVA followed by Tukey’s post-hoc: *p < 0.05, **p < 0.01, ***p < 0.001, ****p < 0.0001 for each group.

Fig. 6 (A, B and C) presents the object recognition indices that provide information on the learning and memory of non-spatial recognition in rats. We noted a significant reduction in short-term IR in the SCO + H_2_O group compared to the Normal Control group (respectively 0.56 ± 0.01; 0.78 ± 0.014 p < 0.0001) (Fig. 6 A). The presence of TT-TeMac™ in the SCO + TT-TeMac™ group significantly increased this IR compared to the SCO + H_2_O group (respectively 0.67 ± 0.04; 0.56 ± 0.01 p < 0.05) (Fig. 6 A). Scopolamine injection in the SCO + H_2_O_2_ group resulted in a reduction of long-term IR compared to the normal control group (respectively 0.53 ± 0.02; 0.72 ± 0.02 p < 0.01) (Fig. 6 B). Co-treatment with TT-TeMac™ (100 mg/kg bw) in the SCO + TT-TeMac™ group showed an increase in this IR compared to the SCO + H_2_O group (0.59 ± 0.03; 0.53 ± 0.02 p > 0.05) (Fig. 6 B). No difference was noted between the SCO + Done group and the SCO + TT-TeMac™ group on this IR (short and long term).

### 3.9 TT-TeMac™ prevents spatial memory deficits in MWM test

**Fig. 7:**
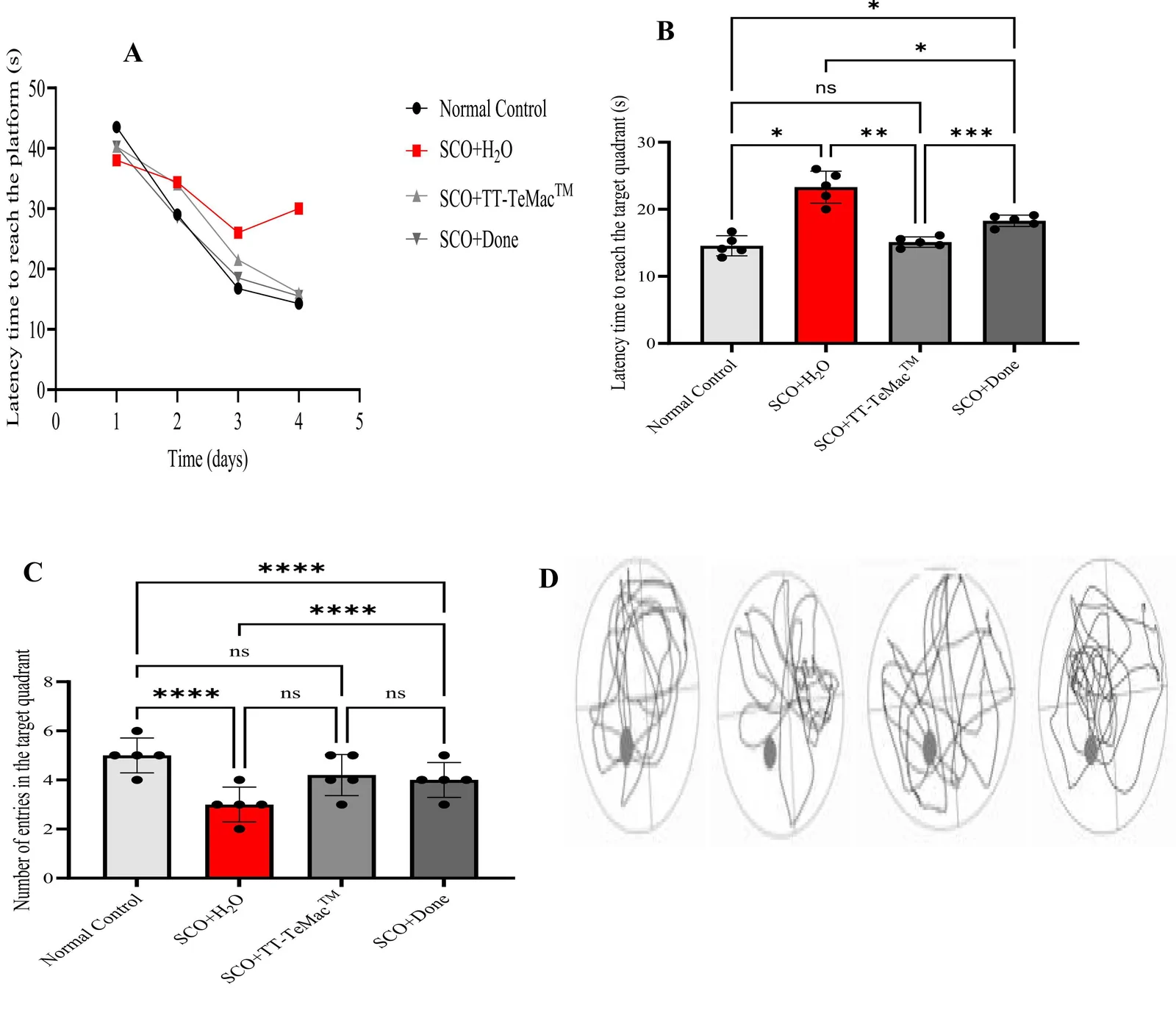
TT-TeMac™ prevents scopolamine-induced spatial learning and memory impairment in rats. *(a) Latency to reach the platform during training days (day 1 to day 4). (B) Latency on the final test day (day 5). (C) Number of entries in the target quadrant. (D) Representative tracks of mice swimming per group during the final test. **Normal Control (n = 5)**: Rats treated with distilled water; **SCO + H***_***2***_***O (n = 5)**: Rats treated with 1 mg/kg body weight/day scopolamine + distilled water; **SCO + TT-TeMac***^***TM***^***(n = 5)**: Rats treated with 1 mg/kg body weight/day scopolamine + 100 mg/kg body weight/day TT-TeMac*^*TM*^*; **SCO + Done (n = 5)**: Rats treated with 1 mg/kg body weight/day scopolamine + 5 mg/kg body weight/day donepezil.* One-way ANOVA followed by Tukey’s post-hoc: *p < 0.05, **p < 0.01, ***p < 0.001, ****p < 0.0001 for each group.

Fig. 7 (A, B and C) shows the learning and memory parameters during MWM. Scopolamine induced learning impairments in the SCO + H_2_O group compared to the normal control group, characterized by a change in the learning profile (Fig. 7A); however, treatment with TT-TeMac™ (100 mg/kg bw) in the SCO + TT-TeMac™ group maintained the learning profile. On the last day of testing, we assessed memory parameters. It appears that scopolamine (1 mg/kg bw) treatment in the SCO + H_2_O group significantly increased the latency time (s) to reach the target quadrant (respectively 23.31 ± 2.38; 14.56 ± 1.49 p < 0.05) and reduced (respectively 3 ± 0.7; 5 ± 0.7 p < 0.0001) the number of entries into the target quadrant compared to the normal control group. In contrast, TT-TeMac™-treated rats in the SCO + TT-TeMac™ group showed improvements in spatial memory, characterized by a reduction (respectively 15.1 ± 0.77; 23.31 ± 2.38 p < 0.01) in the latency to reach the target (s) quadrant and an increase (respectively 4.2 ± 0.83; 3 ± 0.7 p > 0.05) in the number of entries into the target quadrant compared to the SCO + H_2_O group. TT-TeMac™ was comparable to that of donepezil (Fig. 7 B).

### 3.10 TT-TeMac™ protects the loss of neuron on hippocampal microarchitecture rat’s brain

#### 3.10.1 TT-TeMac™ protects the loss of neuron on CA1 hippocampal microarchitecture rat’s brain

Fig. 8a show the effect of TT-TeMac™ (100 mg/kg bw) in CA1 of the hippocampus in rats treated with scopolamine (1 mg/kg bw). Histological analysis with H&E revealed altered structural integrity, with leukocyte infiltration in the CA1 region of the hippocampus in SCO + H_2_O rats compared to the normal control. Administration of TT-TeMac™ in SCO + TT-TeMac™ protected the CA1 region of the hippocampus in these rats from this alteration (absence of leukocyte infiltration). However, the structural morphology of the CA1 region of the hippocampus was similar to that of the SCO + Done group. The use of CV showed that scopolamine caused neuronal apoptosis, thus reducing neuronal density in the CA1 area of the hippocampus of rats in the SCO + H_2_O group compared to rats in the normal control group. The group of rats receiving TT-TeMac™ (SCO + TT-TeMac™) prevented this neuronal apoptosis, thus reducing neuronal loss.

**Fig. 8a:**
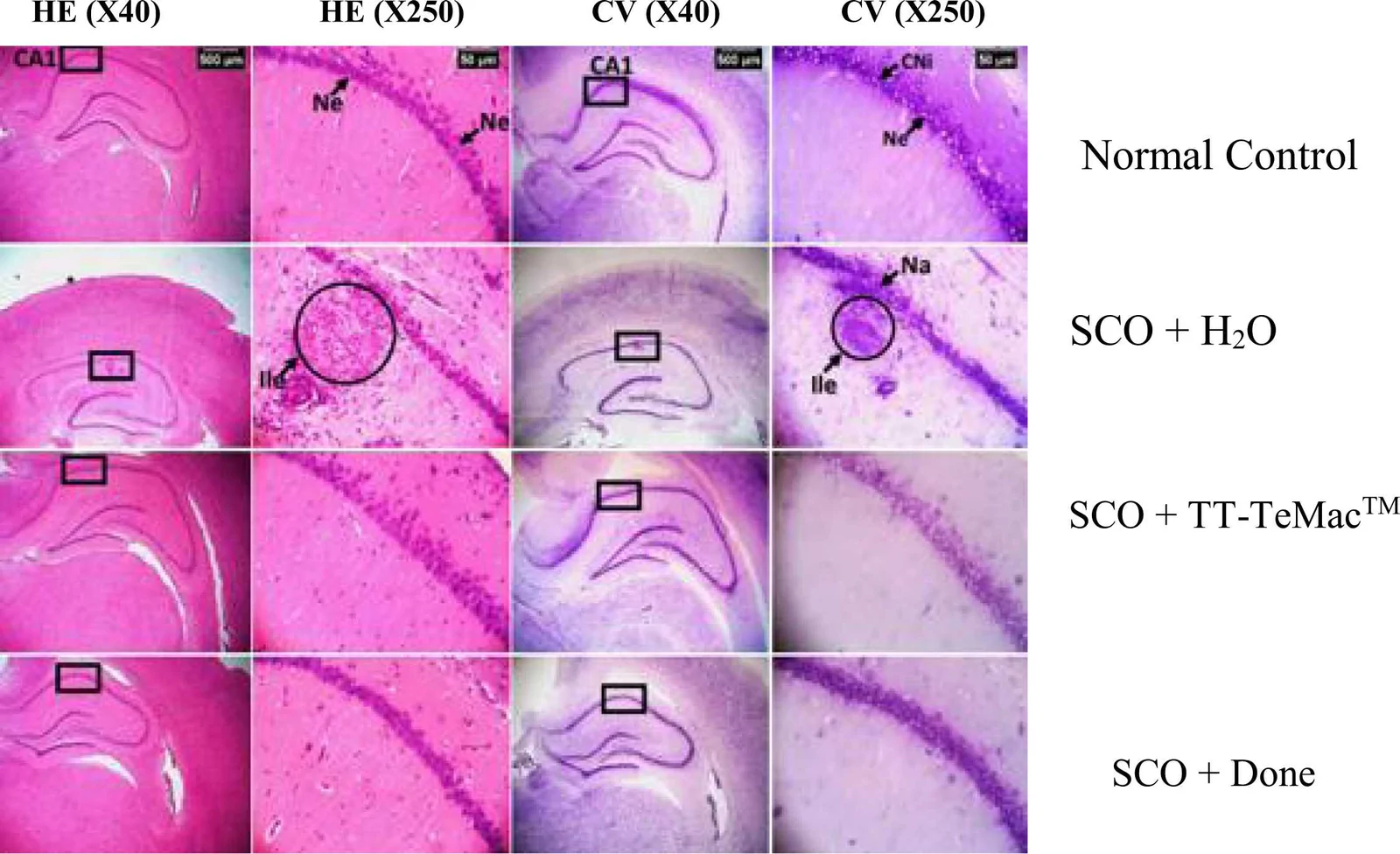
TT-TeMac™ protects loss of neurons in the CA1 of hippocampus of rats. HE = Hematoxylin-eosin; CV = Cresyl violet; Ne = Neuron; Ile = Leukocyte infiltration; Na = Apoptotic neuron; CNi = Nissl body; CA1: Cornu Ammonis 1. **Normal Control (n = 5)**: Rats treated with distilled water; **SCO + H**_**2**_**O (n = 5)**: Rats treated with 1 mg/kg body weight/day scopolamine + distilled water; **SCO + TT-TeMac**^**TM**^**(n = 5)**: Rats treated with 1 mg/kg body weight/day scopolamine + 100 mg/kg body weight/day TT-TeMac™; **SCO + Done (n = 5)**: Rats treated with 1 mg/kg body weight/day scopolamine + 5 mg/kg body weight/day donepezil.

#### 3.10.2 TT-TeMac™ protects the loss of neuron on CA2 hippocampal microarchitecture rat’s brain

Fig. 8b show the effect of TT-TeMac™ in CA2 of the hippocampus in rats treated with scopolamine (1 mg/kg bw). Observation of the CA2 region of the hippocampus in SCO + H_2_O rats, after H&E staining, revealed cell loss compared to the control group. Treatment with TT-TeMac™ reduced this loss. However, the structural morphology of the CA2 region of the hippocampus in SCO + TT-TeMac™ rats was closer to that of the control group than to that of the SCO + Done group. CV-specific staining highlighted hypotrophy and pyknosis in neurons of SCO + H_2_O rats, compared to the control group. Treatment with TT-TeMac™ (SCO+TT-TeMac™) prevented this hypotrophy and pyknosis, thus reducing neuronal loss.

**Fig. 8b:**
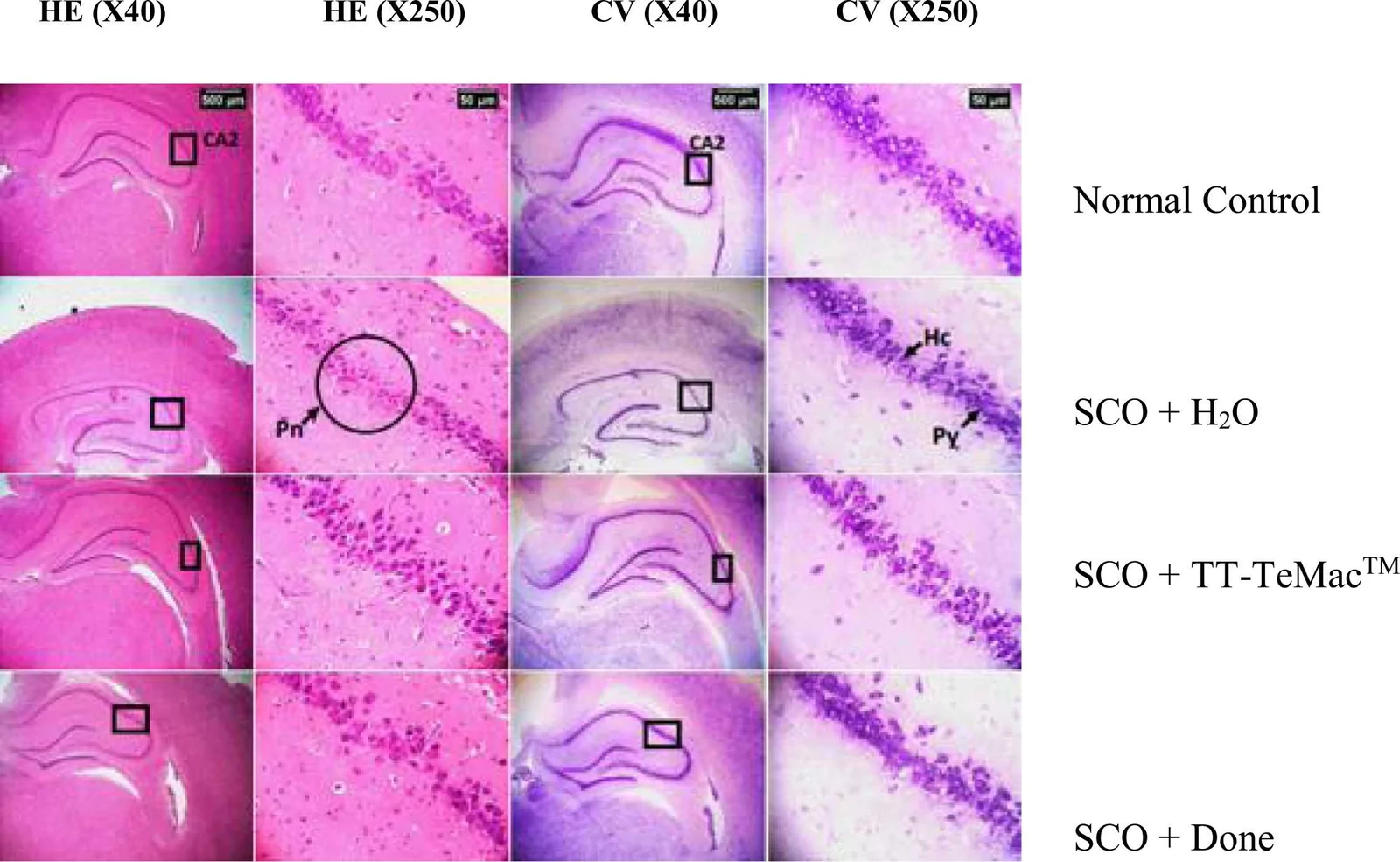
TT-TeMac™ protects loss of neurons in the CA 2 of hippocampus of rats. HE = Hematoxylin-eosin; CV = Cresyl violet;; Pn = Neuronal loss; Hc = Cell hypotrophy; Py = Pycnosi; CA2: Cornu Ammonis 2;**Normal Control (n = 5)**: Rats treated with distilled water; **SCO + H**_**2**_**O (n = 5)**: Rats treated with 1 mg/kg body weight/day scopolamine + distilled water; **SCO + TT-TeMac**^**TM**^**(n = 5)**: Rats treated with 1 mg/kg body weight/day scopolamine + 100 mg/kg body weight/day TT-TeMac™; **SCO + Done (n = 5)**: Rats treated with 1 mg/kg body weight/day scopolamine + 5 mg/kg body weight/day donepezil.

#### 3.10.3 TT-TeMac™ protects the loss of neuron on CA3 hippocampal microarchitecture rat’s brain

Fig. 8c show the effect of TT-TeMac™ in CA3 of the hippocampus in rats treated with scopolamine. Observation of the CA3 region of the hippocampus in rats from the SCO + H_2_O group, after H&E staining, revealed altered morphology and cell loss compared to the control group. Treatment with TT-TeMac™ reduced this loss. However, the structural morphology of the CA3 region of the hippocampus in rats from the SCO + TT-TeMac™ group is closer to that of the control group than to that of the SCO + Done group. CV-specific staining revealed the presence of pyknosis in neurons of SCO + H_2_O rats, compared to the control group. Treatment with TT-TeMac™ (SCO + TT-TeMac™) prevented this hypotrophy and pyknosis.

**Fig. 8c:**
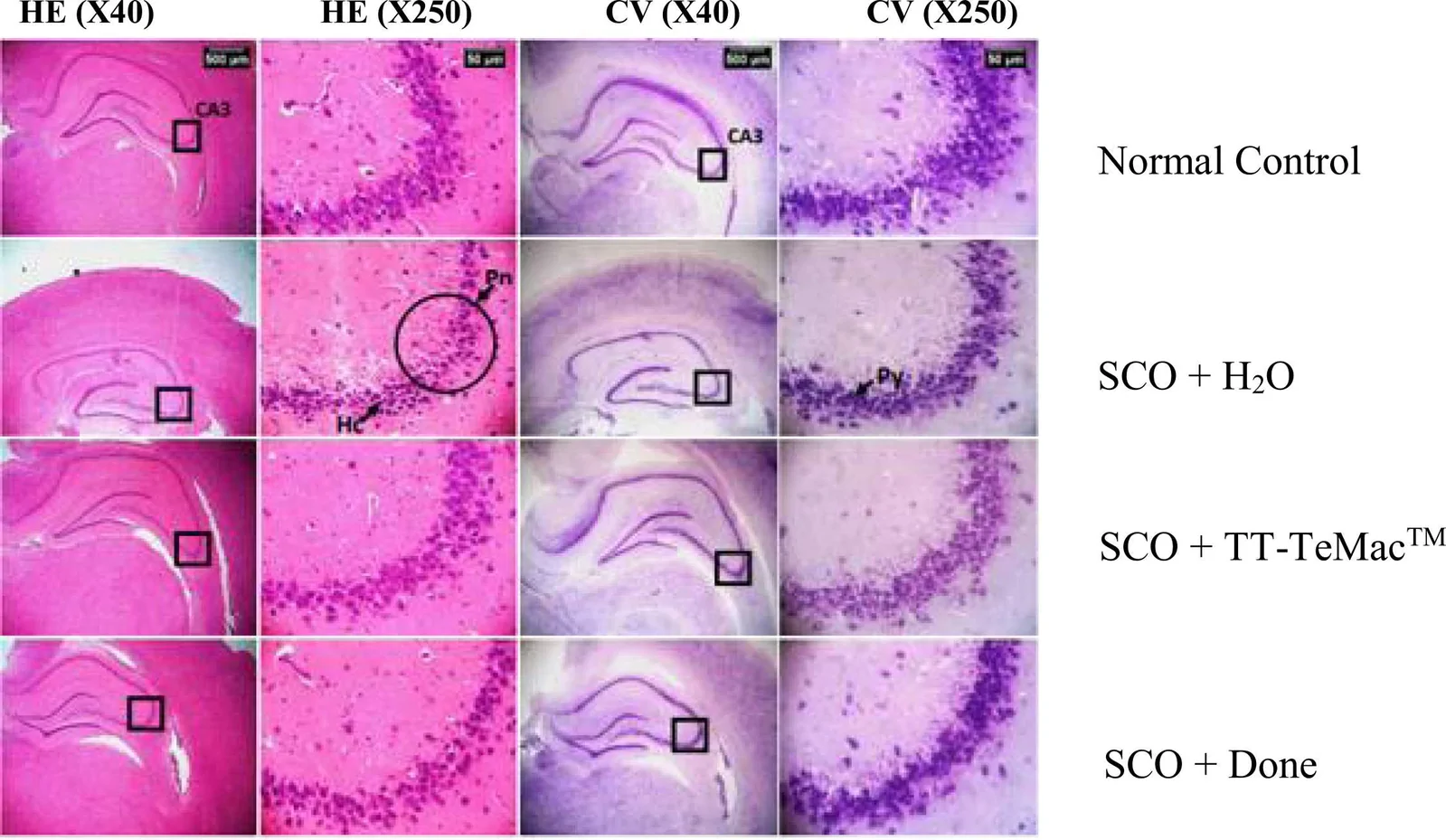
TT-TeMac™ protects loss of neurons in the CA3 of hippocampus of rats. HE = Hematoxylin-eosin; CV = Cresyl violet; Pn = Neuronal loss; Hc = Cell hypotrophy; Py = Pycnosis; CA3: Cornu Ammonis 3; **Normal Control (n = 5)**: Rats treated with distilled water; **SCO + H**_**2**_**O (n = 5)**: Rats treated with 1 mg/kg body weight/day scopolamine + distilled water; **SCO + TT-TeMac**^**TM**^**(n = 5)**: Rats treated with 1 mg/kg body weight/day scopolamine + 100 mg/kg body weight/day TT-TeMac™; **SCO + Done (n = 5)**: Rats treated with 1 mg/kg body weight/day scopolamine + 5 mg/kg body weight/day donepezil.

#### 3.10.4 TT-TeMac™ protects the loss of neuron on GD hippocampal microarchitecture rat’s brain

Fig. 8d show the effect of TT-TeMac™ in GD of the hippocampus in rats treated with scopolamine. Observation of the hippocampal GD region in rats from the SCO + H_2_O group, using H&E staining, showed a morphological alteration with a marked opening of the hippocampal sulcus (or hippocampal fissure) and cellular loss, compared to the control group. Treatment with TT-TeMac™ reduced this loss. However, the structural morphology of the CA3 area of the hippocampus in rats from the SCO + TT-TeMac™ group is closer to that of the normal group than to that of the SCO + Done group.

**Fig. 8d:**
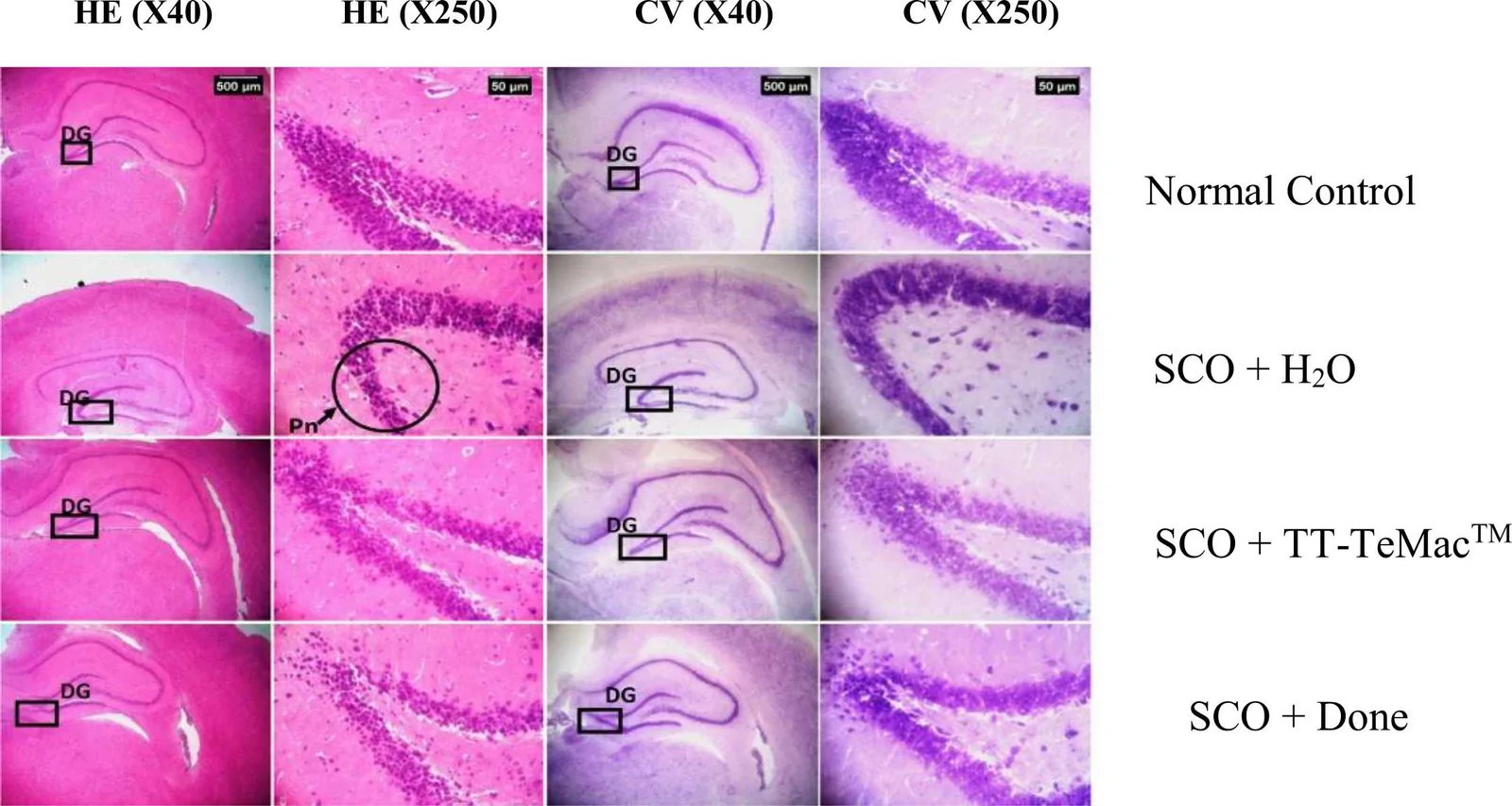
TT-TeMac™ protects loss of neurons in the DG of hippocampus of rats. HE = Hematoxylin-eosin; CV = Cresyl violet; Pn = Neuronal loss; GD = Dentate gyrus; **Normal Control (n = 5)**: Rats treated with distilled water; **SCO + H**_**2**_**O (n = 5)**: Rats treated with 1 mg/kg body weight/day scopolamine + distilled water; **SCO + TT-TeMac**^**TM**^**(n = 5)**: Rats treated with 1 mg/kg body weight/day scopolamine + 100 mg/kg body weight/day TT-TeMac™; **SCO + Done (n = 5)**: Rats treated with 1 mg/kg body weight/day scopolamine + 5 mg/kg body weight/day donepezil.

### 3.11 TT-TeMac™ reduces cholinesterase activity in the brain

**Fig. 9:**
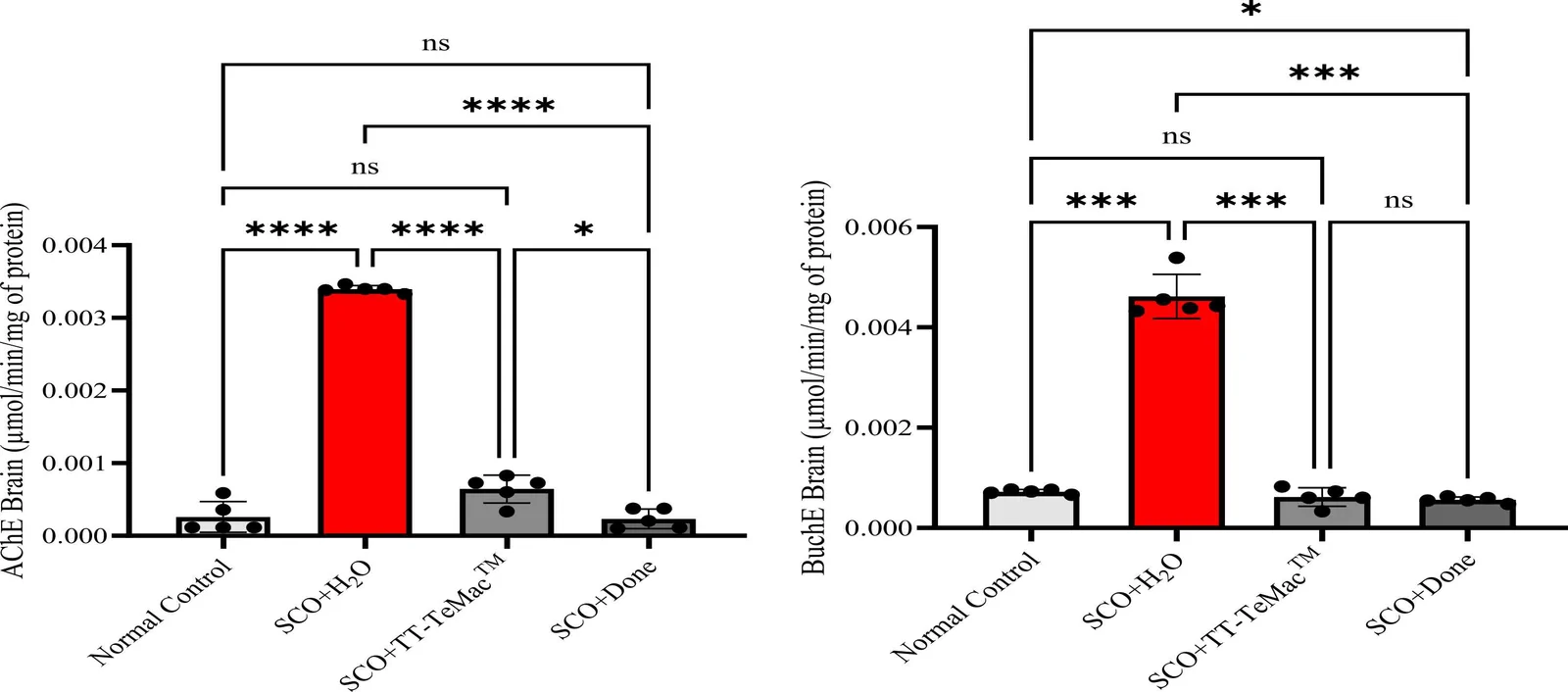
TT-TeMac™ counteracts the increase in brain cholinesterase activities. (a): Acetylcholinesterase (AchE), (b): Butyrylcholinesterase (BuchE). ***Normal Control (n = 5)**: Rats treated with distilled water; **SCO + H***_***2***_***O (n = 5)**: Rats treated with 1 mg/kg body weight/day scopolamine + distilled water; **SCO + TT-TeMac***^***TM***^***(n = 5)**: Rats treated with 1 mg/kg body weight/day scopolamine + 100 mg/kg body weight/day TT-TeMac*^*TM*^; **SCO + Done (n = 5)**: Rats treated with 1 mg/kg body weight/day scopolamine + 5 mg/kg body weight/day donepezil. One-way ANOVA followed by Tukey’s post-hoc: *p < 0.05, **p < 0.01, ***p < 0.001, ****p < 0.0001 for each group.

Acetylcholinesterase activity was significantly increased in the SCO + H_2_O group compared to the normal control group (respectively 0.00339 ± 4.96E−05; 0.000259 ± 0.0002, p < 0.0001) (Fig. 9). In addition, administration of TT-TeMac™ at 100 mg/kg body weight/day in the SCO + TT-TeMac™ group resulted in a significant reduction in this enzyme activity compared to the SCO + H_2_O group (respectively 0.00064 ± 0.0001; 0.00339 ± 4.96E−05, p < 0.001).

In the same direction, the activity of butyrylcholinesterase was significantly higher in the SCO + H_2_O group than in the normal control group (respectively 0.0046 ± 0.0004; 0.00072 ± 0.00004, p < 0.001). Concomitant administration of TT-TeMac™ at 100 mg/kg body weight/day in the SCO + TT-TeMac™ group resulted in a significant reduction in the activity of this enzyme compared to the SCO + H_2_O group (respectively 0.00061 ± 0.00018; 0.00072 ± 0.00004, p < 0.001). An inhibitory effect of TT-TeMac™ similar to that of the reference drug, donepezil, was observed (0.00061 ± 0.00018; 0.0005 ± 5.75E−05 respectively, p > 0.05) (Fig. 9).

## 4 Discussion

Preventing cognitive decline is essential for combating AD, especially in the context where is there is a lack of curative treatment (Tipton, 2024). Memory loss is the main symptom affecting the quality of life of patients with AD, and treatments do not effectively improve memory loss. This is likely due to the single-target action, yet polypharmacology shows promise for complex diseases. Computational approaches, including network pharmacology and molecular docking have proven to be valuable tools to facilitate the exploration of the pharmacological properties of active ingredients against AD. Recently, Ambamba et al., 2025a, Ambamba et al., 2025b, showed that *Terminalia macroptera* modulate cholinergic dysfunction associated with memory loss in scopolamine-treated rats. In this work we explored network pharmacology and molecular docking followed by experimental validation to explore the neuroprotective effects of TT-TeMac™ on cholinergic dysfunction associated with memory loss in scopolamine-treated rats.

The LC-MS analysis of the TT-TeMac™ allowed the identification of seven compounds, namely Terminolic acid, Sericic acid, Arjunolic acid, Gallic acid, Ellagic acid, 3-O methyl ellagic acid and 3,3’-di-O-methyl ellagic acid. These compounds have been previously isolated from this plant by Conrad et al., 1998; Conrad et al., 2001; Adey et al., 2021 and Roméo et al., 2024.

Using network pharmacology analysis makes it possible to identify the key targets of bioactive compounds involved in the pathophysiological mechanism, thus guiding more targeted and cost-effective experimental validation. In this study, the network pharmacology analysis revealed nine pivotal genes that are potential targets of TT-TeMac™ compounds in the treatment of cholinergic dysfunction. These genes include ACHE, IL6, TNF, SNCA, AKT1, SERPINE1, STAT3, ACE, and ALB. Scientific evidence suggests the involvement of all proteins through these genes in cholinergic dysfunction (Madziar et al., 2008; Tyagi et al., 2010; Chen et al., 2022). Since a protein is the functional version of a gene, molecular docking study was done to confirm the targets generated by the network pharmacology. Molecular simulation showed that all priority proteins have a high binding affinity with the identified TT-TeMac™ compounds (with binding energies ranging from −3.39 to −7.40 kcal/mol). In addition to good binding energy, these compounds interact with amino acids in the active pockets of important proteins involved in the pharmacological network (Table 4). These results predict that, TT-TeMac™ compounds have multi-target actions on proteins involved in cholinergic dysfunction.

Scopolamine, which induces a cholinergic deficit, experimentally reproducing memory disorders similar to those observed in dementia, has been used to experimentally validate the neuroprotective effect of TT-TeMac™. During the MWM, the learning profile that was modified by scopolamine was preserved by the treating with TT-TeMac™ (Fig. 7A). The mean escape latency increased significantly in the scopolamine-treated group during the acquisition phase. However, this change was significantly reversed by TT-TeMac™ therapy (Fig. 7 B). The number of times that the animals entered the target quadrant during the tests was used to assess their memory of the exact location where the platform had previously been placed. Rats treated with scopolamine were less present in the target quadrant, suggesting a problem with spatial memory and learning. In contrast, treatment with TT-TeMac™ increased their presence in the target quadrant. Furthermore, TT-TeMac™ increased the time spent exploring the novel object compared to the well-known object and increased the RI in the NOR (Fig. 6).

All of these results show that TT-TeMac™ (100 mg/kg bw) protects against cholinergic dysfunction associated with scopolamine-induced memory loss in rats. This is explained firstly by the ability of TT-TeMac™ to protect certain areas of the hippocampus (CA1, CA2, CA3 and DG) against scopolamine-induced neurodegeneration (Fig. 8a, Fig. 8b, Fig. 8c, Fig. 8d) and secondly by preventing the increase in cholinesterase activity (AChE and BuchE) (Fig. 9). The reduction in AChE activity validates the results of molecular docking, which showed that the compounds in TT-TeMac™ have an affinity for AChE and interact with Trp86, Asp74, Tyr341, and Tyr124 residues present in various acyl and anionic pockets. Furthermore, one of the ingredient's components (arjunolic acid) interacts with His447, which is involved in the catalytic triad of the functional AChE mechanism.

## 5 Conclusion and Future Directions

This study demonstrates that TT-TeMac™, through its bioactive compounds (terminolic acid, sericic acid, arjunolic acid, gallic acid, ellagic acid and its derivatives), targets 15 genes common to cholinergic dysfunction. Network pharmacology and molecular docking confirmed the strong interaction between the compounds and key genes of the cholinergic pathway. Moreover, *in vivo* validation revealed that TT-TeMac™ effectively counteracts scopolamine-induced memory impairment by inhibiting cholinesterase activity and preserving hippocampal neuronal integrity. These results suggest that TT-TeMac™ has promising therapeutic potential for the management of cognitive decline in AD. Given the multifactorial nature of AD, the multitarget action of TT-TeMac™ highlights its advantage over single-target therapies, potentially offering a more comprehensive approach to mitigating neurodegeneration. Future studies should validate its effects on cellular models of cholinergic dysfunction, thus better highlighting the mechanisms of action.

## CRediT authorship contribution statement

**Messanga me Ngo'o Jonathan:** Methodology, Formal analysis, Data curation. **Akono Fama Yves Marc:** Methodology, Formal analysis, Data curation. **Damaris Enyegue Mandob:** Writing – review & editing, Supervision, Methodology. **Judith Laure Ngondi:** Writing – review & editing, Supervision, Project administration, Conceptualization. **AMBAMBA AKAMBA Bruno Dupon:** Writing – review & editing, Writing – original draft, Visualization, Validation, Methodology, Formal analysis, Data curation, Conceptualization. **Njayou Mbouangouore Ingrid Reine:** Methodology, Formal analysis, Data curation. **Fils Armand Ella:** Writing – review & editing, Methodology, Formal analysis. **alexandra ebogo Enyegue Françoise:** Methodology, Formal analysis, Data curation. **Nkodo Abega Laurent:** Visualization, Formal analysis, Data curation. **la blonde njanjo Ejanmoua Merveille:** Methodology, Formal analysis, Data curation. **Nyabissick Mondjiep Sandrine:** Methodology, Formal analysis, Data curation. **Ngarchindi Emmanuel:** Methodology, Formal analysis, Data curation.

## Declaration of Competing Interest

The authors declare that they have no known competing financial interests or personal relationships that could have appeared to influence the work reported in this paper.
