# Supplementary material for: Network pharmacology, molecular docking and In vivo validation reveal the neuroprotective effects of TT-TeMac™ against cholinergic dysfunction and hippocampal lesions
Source: IBRO Neurosci Rep. 2026 Aug 10;21:568–81. doi: 10.1016/j.ibneur.2026.08.007 (PMC13499058; doi:10.1016/j.ibneur.2026.08.007)
Supplement: Supplementary file 1 [file mmc1.docx]

| Compounds   a) **Supplementary material** : 2D structural pose of the interactions between TT-TeMac^TM^ compounds and targets | ACHE | ACE | ABL | STAT3 | SERPINE1 |
| --- | --- | --- | --- | --- | --- |
| Terminolic Acid |  |  |  |  |  |
| Sericic acid |  |  |  |  |  |
| Arjunolic acid |  |  |  |  |  |
| Gallic Acid |  |  |  |  |  |
| Ellagic Acid |  |  |  |  |  |
| 3-O-methyl ellagic acid |  |  |  |  |  |
| 3,3’-di-O-methyl ellagic acid |  |  |  |  |  |

| Compounds | AKT1 | SNCA | TNF | IL6 |
| --- | --- | --- | --- | --- |
| Terminolic Acid |  |  |  |  |
| Sericic acid |  |  |  |  |
| Arjunolic acid |  |  |  |  |
| Gallic Acid |  |  |  |  |
| Ellagic Acid |  |  |  |  |
| 3-O-methyl ellagic acid |  |  |  |  |
| 3,3’-di-O-methyl ellagic acid |  |  |  |  |

**b) Supplementary material:** RMSD between the compounds of TT-TeMac^TM^ and prioritized cholinergic dysfunction target proteins

| Compounds | ACHE | ACE | ABL | STAT3 | SERPINE1 | AKT1 | SNCA | TNF | IL6 |
| --- | --- | --- | --- | --- | --- | --- | --- | --- | --- |
| Terminolic Acid | 1.61 | 1.72 | 1.79 | 1.85 | 2.02 | 1.57 | 1.84 | 1.53 | 1.22 |
| Sericic acid | 1.64 | 1.36 | 1.51 | 1.64 | 1.94 | 1.96 | 1.64 | 1.17 | 1.39 |
| Arjunolic acid | 1.86 | 1.79 | 1.42 | 1.78 | 1.58 | 1.49 | 1.75 | 1.68 | 1.52 |
| Gallic Acid | 0.61 | 0.64 | 1.09 | 1.94 | 1.62 | 1.27 | 1.24 | 1.61 | 1.84 |
| Ellagic Acid | 1.85 | 1.20 | 1.32 | 1.25 | 1.68 | 1.61 | 1.36 | 1.38 | 1.64 |
| 3-O methyl ellagic acid | 1.11 | 2.04 | 0.94 | 1.37 | 1.49 | 1.78 | 1.28 | 1.54 | 1.54 |
| 3,3’-di-O-methyl ellagic acid | 0.72 | 1.83 | 1.74 | 1.43 | 1.78 | 1.65 | 1.94 | 1.24 | 1.67 |
